# Reply to the letter regarding ‘Unravelling the genomic landscape of Canadian Borrelia burgdorferi’

**DOI:** 10.1099/mgen.0.001678

**Published:** 2026-05-11

**Authors:** Anthony Piot, Iain L. Mainprize, Justin Wood, Jeff Gauthier, Cezar M. Khursigara, Karine Thivierge, Melanie K. B. Wills, Roger C. Levesque

**Affiliations:** 1 Institut de Biologie Intégrative et des Systèmes (IBIS), Université Laval, Québec City, Québec, Canada; 2 Faculté de médecine, Université Laval, Québec City, Québec, Canada; 3 Department of Molecular and Cellular Biology, G. Magnotta Lyme Disease Research Lab, University of Guelph, Guelph, Ontario, Canada; 4 Department of Molecular and Cellular Biology, University of Guelph, Guelph, Ontario, Canada; 5 Laboratoire de santé publique du Québec, Québec, Canada; 6 Institute of Parasitologie, McGill University, Sainte-Anne-de-Bellevue, Canada

**Keywords:** *Borrelia*, comparative genomics, genome assembly, hybrid assembly, long reads

Following the publication of our article on the comparative genomics of Canadian *Borrelia burgdorferi* strains [1], Ogden and colleagues provided thoughtful comments about the results of our study, to which we would like to offer some clarifications.

## Genome completeness and plasmid reconstruction

Despite technological and methodological advances, reconstruction of the *Borrelia* plasmid complement remains technically challenging. The authors of the letter raise concerns that the genomes we produced might be incomplete, based on the bioinformatics methods employed and comparisons with reference isolates. Our assemblies were generated using long and short sequencing data with careful evaluation and cross-evidence from multiple common metrics for assessing genome completeness and assembly accuracy, including read coverage, telomere identification, plasmid circularization and similarity with reference replicons, as well as gene and *k-mer* completeness [2,3]. The rare cases in which these assembly metrics suggested uncertainties about the completeness of genomes reconstructed with our assembly pipeline are highlighted in our manuscript. Plasmid instability associated with isolate handling and passage history is another challenge that complicates comparison with reference strains [4,5]. Our hybrid assemblies are intended to improve the structural resolution of plasmids compared with short-read-only approaches rather than to assert that every historical plasmid is necessarily retained in cultured isolates. We, therefore, consider our assemblies to represent the plasmid complements of the isolates sequenced under the conditions studied, while acknowledging that plasmid composition in *Borrelia* may vary among laboratory-propagated strains, as discussed in our study.

## Genomic resources for Canadian *Borrelia* strains

Previous MLST-based and whole-genome studies of Canadian and North American *B. burgdorferi* populations have been foundational contributions to the field [6,9]. Our study did not aim to overlook the impact of previous work on Canadian *Borrelia* strains, but rather to emphasize that genomic resources available for Canadian isolates are limited comparatively to other regions. A fact exemplified by the absence of a complete Canadian *Borrelia* genome, including plasmids, in the scientific literature and public databases at the time.

## Interpretation of phylogenetic inferences

Comparison between the phylogenies we obtained from chromosomal multi locus sequence typing (MLST) (using eight housekeeping genes) and whole-genome markers for *B. burgdorferi* strains presents important topological differences. The letter’s authors criticize our claim that topological differences between the two phylogenies ‘indicated that limited gene marker sets are not representative of the full evolutionary history of the bacteria’ and they stated in response that ‘for the most part clades are stable across trees (i.e. strains that cluster together remain grouped), and what changes is mainly the relative placement of those clades and a few deeper/internal nodes’. We can only disagree with this dismissal of the importance of relative clade placement in evolutionary analyses. Relative clade placement not only constitutes one of the basis of cladistics but is also a crucial result of any phylogenetic inference informing interpretations of evolutionary history, ancestry, and divergence. While strain clustering within clades may be broadly concordant, differences in deeper topology lead to divergent interpretations of evolutionary history. MLST remains a useful method for what it is meant to be, an isolate typing tool [10]. However, our study and previous work by the letter’s authors [8] demonstrate that MLST and chromosome-wide phylogenies present large topological differences, indicative of different evolutionary scenarios. These discrepancies reflect the increased resolution afforded by genome-wide data [11]. We, therefore, consider it reasonable to argue that MLST and its low marker density are inferior to chromosome-wide phylogenies for studying *Borrelia* evolutionary history.

## Scope of global comparison

Another comment notes that only 13 strains have been explored in our study, which is incorrect. While we sequenced and assembled only 13 *Borrelia* genomes, we used every *B. burgdorferi* complete genome available on the NCBI's Genome database (www.ncbi.nlm.nih.gov/datasets/genome) to perform comparative genomics and phylogenetic inferences with Canadian strains. This broader comparative context enabled us to increase the resolution required to study the evolution and genomic structure of these strains. All the methods for these analyses are detailed in our manuscript.

## Conclusion

We consider the approach presented in our study and the associated findings as complementary to prior work and as an incremental methodological advance for *Borrelia* genome reconstruction and research. This strategy has the potential to facilitate more comprehensive characterization of the full plasmid complement of Borrelia genomes, which have historically been difficult to resolve and continue to pose technical challenges. We appreciate the opportunity to clarify some aspects of our study and welcome continued discussion as genomic tools for investigating this complex pathogen rapidly evolve.

## Abbreviations

MLST: multi locus sequence typing
NCBI: National Center for Biotechnology Information
